# Quantitative proteomics analysis of proteins involved in alkane uptake comparing the profiling of *Pseudomonas aeruginosa* SJTD-1 in response to n-octadecane and n-hexadecane

**DOI:** 10.1371/journal.pone.0179842

**Published:** 2017-06-29

**Authors:** Xuefeng Zhou, Xuejiao Xing, Jingli Hou, Jianhua Liu

**Affiliations:** 1 School of Life Science & Biotechnology, Shanghai Jiao Tong University, Shanghai, China; 2 School of Pharmacy, Shanghai Jiao Tong University, Shanghai, China; 3 Instrumental Analysis Center of Shanghai Jiao Tong University, Shanghai, China; University of Glasgow, UNITED KINGDOM

## Abstract

While many data are available on genes encoding proteins for degradation of hydrocarbons in bacteria, the impact of alkane on transporter protein expression is unclear. *Pseudomonas aeruginosa* SJTD-1 is a strain that can consume medium- and long-chain n-alkanes. In order to study the proteins involved in n-octadecane uptake, we use iTRAQ and label free comparative proteomics analysis to identify the proteins of alkane uptake in response to n-octadecane (C18) comparing with n-hexadecane (C16) in *P*. *aeruginosa* SJTD-1. A total of 1102 and 1249 proteins were identified by iTRAQ-based and label free quantitative methodologies, respectively. By application of 1.5 (iTRAQ) or 2-fold (label free) for upregulated and 0.65 (iTRAQ) or 0.5-fold (label free) for downregulated cutoff values, 91 and 99 proteins were found to be differentially expressed comparing SJTD-1 cultivated on C18 with C16 respectively. There are six proteins with the common differential expression by iTRAQ and label free-based methods. Results of bioinformational analysis suggested the involvement of bacterial chemotaxis in responds to C18. Additionally, quantitative reverse transcriptase PCR (qRT-PCR) results confirmed C18-induced change in levels of FleQ, FliC, NirS, FadL and FadD proteins and the role of the proteins in n-octadecane uptake was further discussed in *P*. *aeruginosa*. In conclusion, results of the present study provided information about possible target-related proteins of bacterial chemotaxis, swimming performance, alkane transport to stimulus of n-ctadecane rather than n-hexadecane in *P*. *aeruginosa* SJTD-1.

## Introduction

Some microorganisms are able to use hydrocarbons, although are toxic to animals and plants, as carbon and energy sources [1, 2]. Among multiple hydrocarbon pollutants, *n*-alkanes are a large class of saturated hydrocarbons with extremely low water solubility. Many strains, for example *Alcanivorax*, *Thalassolituus*, *Pseudomonas*, *Rhodococcus*, or *Acinetobacter*, have been found possess the ability to degrade short- (C4-C8), medium- (C5–C16) and long-chain (C10-C36) linear n-alkanes [3–6]. Among them, *Pseudomonads* have been surveyed for their efficient utilization of n-alkanes for a long time. A strain of *P*. *aeruginosa* SJTD-1 was isolated from oil-contaminated soil; its hydrocarbon utilization capability and n-alkane breakdown efficiency were investigated [7].

Many genes encoding proteins for degradation of hydrocarbons has been studied [1,8], yet the proteins involved in the alkane uptake have not been much worked out. The mechanism of alkanes enter the cell may differ depending on the bacterial species considered, the molecular weight of the alkane and the physico-chemical characteristics of the environment [9,10]. It has been shown that the short alkane can be acquired directly by bacteria due to the high water solubility [11,12]. However, the water solubility of n-alkanes decreases exponentially as their molecular weight increases [13], and long-chain alkanes are more persistent in the environment than shorter-chains. It is summarized by Rojo that for medium- and long-chain-length n-alkanes, microorganisms may gain access to them either by adhering to hydrocarbon droplets or by a surfactant-facilitated process [1]. Biofilm formation at the hydrocarbon-water interface has been observed with various alkane-degrading strain including *Pseudomonas sp*. strain 8909N [14,15], and a strong link between biofilm formation and the utilization of alkanes has been indicated in *Marinobacter hydrocarbonoclasticus* SP17 [16]. Hemamalini and Khare presented that the outer membrane porins showed significant differential expression in presence of alkanes [17]. AlkL in *Pseudomonas putida* GPo1, was identified as the first known bacterial alkane importer [18]. The FadL membrane transport was firstly characterized in *E*. *coli* to specifically recognize and transport a long-chain fatty acids (LCFAs) [19,20]. Recently, FadL was also confirmed to server as the major route for medium-chain alkane import in *E*. *coli*.[21]. In our previous study, we use iTRAQ strategy firstly to compare the proteomes of *P*. *aeruginosa* SJTD-1 degrading alkane [22]. Our previous results indicate FadL and type VI secretion system (T6SS) subunits might play significant roles in alkane uptake in *P*. *aeruginosa*.

Although many possible proteins related with alkane assimilation have been identified, the information of alkane uptake offered by the results is limit in *P*. *aeruginosa*, and the passage of proteins of transport and their regulator is still unclear. Hence, it seems quite worthwhile to investigate the proteome profile of *P*. *aeruginosa* cultivated on C18 comparing with C16, especially to see which proteins get differentially expressed in response to n-octadecane and thus play a role in the cellular alkane uptake.

In this case, we used iTRAQ and label free comparative proteomics to characterize the n-octadecane uptake comparing with n-hexadecane in *P*. *aeruginosa* SJTD-1, 91 and 99 proteins were found to been differentially expressed respectively. Additionally, the overexpression of FleQ, FliC, NirS and FadL was confirmed by qRT-PCR in transcript level. In this sense, results of the present study provided information about possible target-related proteins of alkane uptake and of course be important for understanding alkane use in *P*. *aeruginosa* SJTD-1.

## Materials and methods

### Strains and culture media

*P*. *aeruginosa* SJTD-1 was cultured at 37°C with a shaking speed of 200 rpm. Luria-Bertani medium (LB) and C18 or C16 medium containing (K2HPO4 3.815 g, KH2PO4 0.5 g, (NH4)2HPO4 0.825g, KNO3 1.2625 g, Na2SO4 0.2 g, CaCl2 0.02 g, FeCl3 0.002 g, MgCl2 0.02 g/l and 500 mg/l n-octadecane or n-hexadecane) were used in this study.

### Bacteria cultivation and protein preparation

*P*. *aeruginosa* SJTD-1 was precultured overnight in LB at 37°C with vigorous shaking followed by being harvested by centrifugation and washed three times with 1× PBS buffer. The obtained precultures were inoculated to two flasks containing 100 ml C18 or C16 respectively and be cultured as the described above. The C16-grown cells were been selected as the control group in both iTRAQ-based and label-free analysis. Cells of the late exponential phase was harvested after SJTD-1 has been cultivated for 30 hours in alkane medium (OD600 = 0.5~0.8) and re-suspended into the pre-cooled lysis buffer (7 M Urea, 4% CHAPS, 1mM PMSF, 2mM EDTA, 0.5 mM EGTA, 40 mM Tris-HCl, pH 7.4). Sonication was used to shear the cells, and cell debris was removed by centrifugation at 15,000 rpm for 20 min at 4°C. The supernatant was quantified using the Bradford method [23] and store at -80°C.

### Protein digestion and iTRAQ labeling

Each sample of 200 μg protein was reduced using 2 μl 1M DTT at 60°C for 1 h. Cysteine residues were blocked using 10 μl 1M iodoacetamide (IAM) for 10 min at room temperate. Proteins were digested with Sequencing Grade Modified Trypsin (Promega, USA) via the FASP protocol with spin ultrafiltration units of nominal molecular weight cutoff 10,000 [24]. Protein digestions were conducted overnight with trypsin in a 1:25 trypsin-to-protein mass-ratio. After digestion, the liberated peptides were collected by centrifugation and the filtration units were washed with 50 μL of dissolution buffer two times. Samples were labeled using 4plex iTRAQ reagent kit (AB Sciex, Framingham, MA) as follows: Sample C18-1:115; C16-1:117; C18-2:115; C16-2:117; C18-3:116; C16-3:114. Sample C18-1 and C16-1, C18-2 and C16-2, or C18-3 and C16-3, then pooled together respectively and dried by SpeedVac.

### High pH reverse phase liquid chromatography

The peptide mixture was re-dissolved with the buffer A (20 mM ammonium formate, pH10.0), and then fractionated on a Survey HPLC system (Thermo scientific, Wilmington, DE, USA) using a reverse phase column (Agela, Durashell-C18 Column, 2.1 mm × 250 mm, 5 μm, 100 Å). The peptides were eluted with gradient 5~15% B (20 mM ammonium formate in 80% ACN, pH 10.0) in 25min, 15~38% B in 15min, 90% B hold for 10 min at a constant flow rate of 0.8 ml/min. The absorbance at 214 nm was monitored, and a total of 38 fractions were collected and then combined into 9 fractions based on peak intensities.

### One-dimensional gel electrophoresis and In-gel digestion

Four replicates of concentrated *P*. *aeruginosa* SJTD-1 on C18 or C16 were separated on 10% SDS-PAGE gel, and stained with Coomassie Blue. After extensive decolorization, each gel lane was excised into 8 sections. Each excised section was destained by incubation in 50% acetonitrile in 50 mM ammonium bicarbonate. After destained, the gel pieces were reduced by incubation in a solution of 10 mM DTT in 25 mM ammonium bicarbonate at 60°C for 20 min. For alkylation of proteins, the gel was incubated in a solution of 100 mM IAM at room temperature for 15 min, followed by dehydrating in 100% acetonitrile for 15 min. After gel pieces were completely dried by SpeedVac, the pieces were swollen in 100μl of 25 mM ammonium bicarbonate buffer containing 0.01 mg/ml trypsin (Promega, Madison, WI) and incubated overnight at 37°C. Peptides were extracted with 60% acetonitrile containing 5% formic acid and 100% acetonitrile successively, dried by SpeedVac, and stored at -20°C for further analysis.

### Nano LC-MS/MS

The digested peptides were analyzed using an LC system (Nano Pump, Ultimate 3000, Dionex, Thermofisher) coupled with an ESI-Q-TOF mass spectrometer (maXis, Impact, Bruker Daltonik, Germany). Each peptide sample was re-dissolved in 2% acetonitrile with 0.1% formic acid, and then loaded onto a Peptide trap column (0.75 mm*2 cm, 3 μm, Dionex, Thermofisher) with the autosampler of the LC system. To desalt and concentrate the sample, the trap column was washed with 2% acetonitrile and 0.1% formic acid at a flow rate of 5μl/min for 10 min. Then the trapped peptides were released and separated in a C18 capillary column (75 μm*15 cm, 3 μm, Dionex, Thermofisher). The peptides were separated using a solvent system with solvent A consisting of 99.9% water and 0.1% formic acid, and solvent B consisting of 99.9% acetonitrile and 0.1% formic acid. The peptides were eluted with gradient 2~30% B in 80 (iTRAQ) or 60 min(label free), 30~80% B in 10 min, and 80% B hold for 10 min with a constant flow rate of 400 nl/min. The LC setup was coupled online to a Q-TOF using a nano-ESI source (Bruker Daltonik, Germany) in data dependent acquisition mode (m/z 350–1500). The Source Capillary was set at 2000~2400 v, the flow and temperature of dry gas was 2.0 L/min and 150°C respectively. The mass spectrometer was set as one full MS scan followed by ten MS/MS scans on the ten most intense ions from the MS spectrum with the following dynamic exclusion settings: exclusion duration = 15 s.

### Data analysis of iTRAQ-based proteomic analysis

The data analysis method of iTRAQ-based proteomic analysis was the same as the mentioned before [22]. The database being used to search is the NCBI_SJTD1_20140411 database (5618 entries) which was established as the mentioned before [22]. Scaffold Q+ (version_4.4.0, Proteome Software Inc., Portland, OR) was used to quantify the isobaric tag peptide and protein identities. Peptide identifications were accepted if they could be established with probability greater than 81.0% to achieve an FDR less than 1.0% by the Scaffold Local FDR algorithm. Protein identifications were accepted if they could be established at greater than 99.0% probability to achieve an FDR less than 1.0% and contained at least 2 identified peptides. Proteins that contained similar peptides and could not be differentiated on the basis of MS/MS analysis alone were grouped to satisfy the principles of parsimony. Proteins sharing significant peptide evidence were grouped into clusters. Acquired intensities in the experiment were globally normalized across all acquisition runs. Individual quantitative samples were normalized within each acquisition run. Intensities of peptide identification were normalized within the assigned protein. The reference channels were normalized to produce a 1:1 fold change. All normalization calculations were performed using medians to multiplicatively normalize data. Permutation Test (P-Value) Test analysis was performed to examine the biological reproducibility (*P*≤ 0.05). The final list of protein ratios was an average of the protein ratios (115-1/117-1, 115-2/117-2 and 116-3/114-3).

### Data analysis of label free-based proteomic analysis

Quantification of these proteins was performed by a label free approach using the fractionation workflow implemented in the Progenesis LC-MS software [25]. The acquired MS raw data were loaded into Progenesis LC-MS (version 4.1, Nonlinear Dynamics, Newcastle, U.K.), and label free quantification was performed for each condition after peak detection, automatic retention time calibration, and normalization in Progenesis LC−MS. The peaklist was created by Progenesis LC-MS and searched in Mascot (version 2.4.1, Matrixscience, London, U.K.). The NCBI_SJTD1_20140411 database (5618 entries) was the same as mentioned above and the decoy option was selected in Mascot. The following parameters were considered for the searches: peptide mass tolerance was set to 40 ppm, fragment mass tolerance was set to 0.05 Da, and a maximum of two missed cleavage of trypsin was chosen. Carbamidomethyl (C) was set as fixed modification, and oxidation (M), deamidation (NQ) were set as variable modifications. All search results were filtered by 1% FDR by Mascot Percolator algorithm and at least two identified unique peptides for each protein. All highly confident identified peptides were exported and quantified in Progenesis LC−MS. Finally, we considered a fold change ≥2 or ≤0.5 and ANOVA p-value ≤ 0.05 in protein expression as a threshold of biological effect.

### Enrichment and protein-protein interaction network analysis

GO classifications and the pathway analysis of the proteins involved in metabolic process are obtained using PANTHER (Version 11.0, Protein Analysis THrough Evolutionary Relationships, http://pantherdb.org), a widely used online resource for comprehensive protein evolutionary and functional classification. Protein-protein interaction network and KEGG pathways analysis for the differential expression proteins were built using the Search Tool for the Retrieval of Interacting Genes/Proteins (STRING, version 10.0) (accessed at September 20, 2016) with a medium confidence level (0.4) and all available prediction methods (http://string-db.org/) [26].

### RNA extraction and quantitative reverse transcription-PCR (RT-qPCR)

*P*. *aeruginosa* SJTD-1 was cultured to the late exponential phase (OD = 0.5~0.8) as described in “Materials and Motheds” above, and the alkanes were the sole carbon sources. RNA was isolated using the RNA Bacteria Protect Reagent (TaKaRa, Dalian, China). RNA yield was estimated using a NanoDrop UV spectrometer (Thermo Scientific, Wilmington, DE, USA). About 1μg of RNA was reversely transcribed using PrimeScript^TM^ RT Master Mix (TaKaRa, Dalian, China). Quantitative real-time PCR was performed using StepOnePlus Real-Time PCR System (Applied Biosystem, USA) with SYBR Green mix (TaKaRa, Dalian, China) and gene-specific primers (F and R). 16S rRNA was used as reference gene of this quantitative system. The qRT-PCR procedure carried out was as follows: 30 seconds of pre-denaturing, 40 cycles of 95°C for 10 s; 58°C for 30 s; and 72°C for 30 s; followed by a melting curve stage from 58°C to 95°C. The relative fold change in mRNA quantity was calculated using the DDCt method [27]. The cDNA amplification efficiency of the samples and internal standards (16S rRNA) were equivalently modulated. At least three independent quantitative real-time PCR experiments were conducted for each RNA sample. All primers which were used in this research were shown in the S1 Table.

## Results

### Proteins identified in iTRAQ-based and label-free analysis

**Fig 1:**
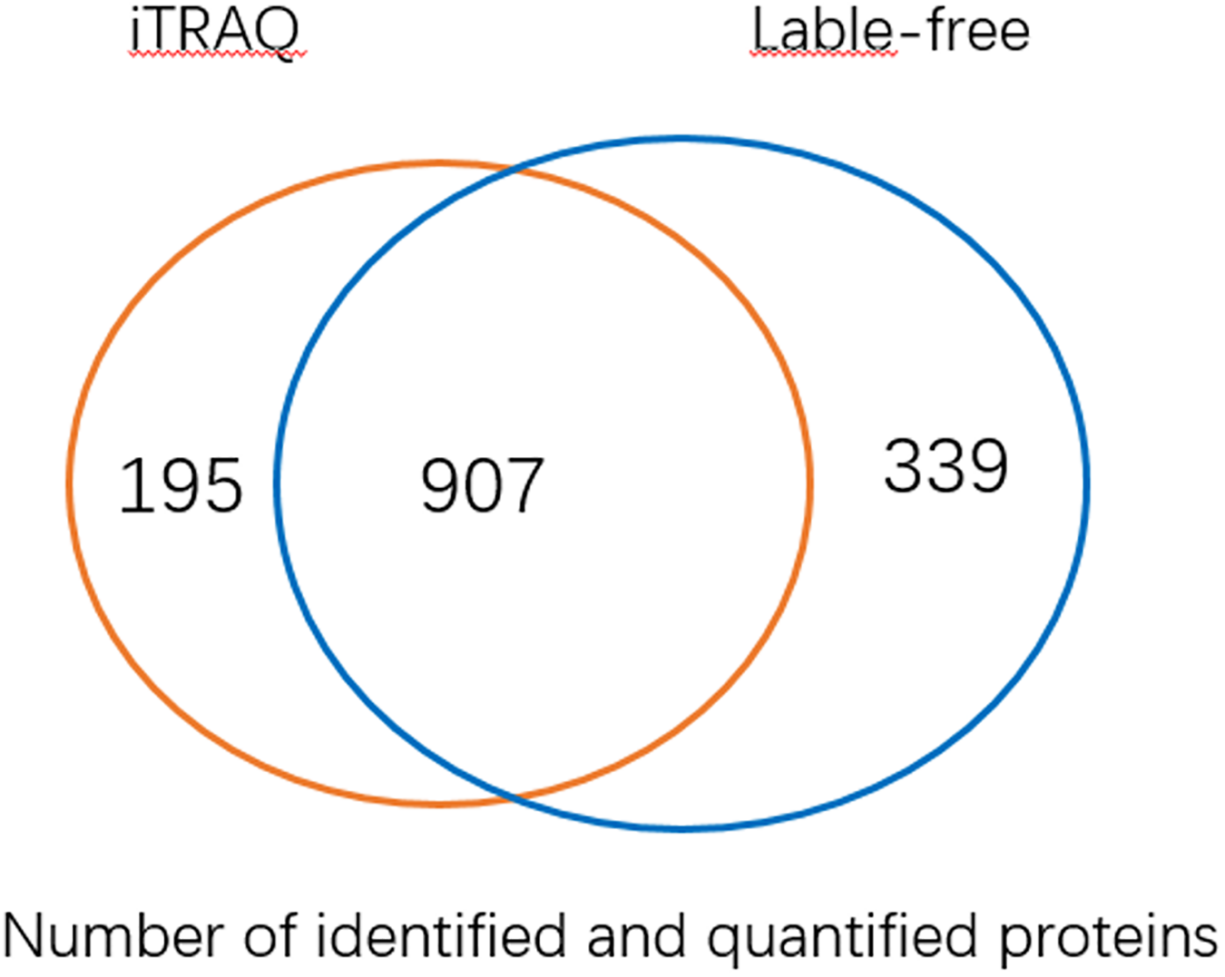
Comparison of results obtained from iTRAQ and label free-based analysis. Number of proteins identified in iTRAQ or label free-based analysis.

In iTRAQ-based analysis, 1102 proteins were been identified and quantified, and totally 91 proteins showed a significant change in expression comparing with control, including 57 being upregulated and 34 downregulated, in C18-cultivated strain (S2 and S3 Tables). In the label-free analysis, 1249 proteins are been identified and quantified and totally 99 proteins showed a significant change, including 89 being upregulated and 10 downregulated, in C18-cultivated strain (S4 and S5 Tables). As shown in Fig 1, by comparing results from iTRAQ and label free-based analysis, we identified 907 proteins in common for both analysis. A total of 184 proteins showing a significant change in expression comparing with control were identified by iTRAQ and label free-based analysis with C18-cultivated strain, and six common proteins showing differential expression were found to be overlapping between the two approaches, of which five proteins were been upregulated. The names of the six common proteins with differential expression and their comparison of the expression levels (ratio between cells cultivated on C18 and C16) were listed in Table 1.

**Table 1:** Identification of common target proteins differentially expressed during *n-*octadecane growth by iTRAQ and label free-based methods^a^.

Accession Number ^b^	Gene Product ^c^	NCBI Locus ID_PAO1	Gene name	Ratio ^d^ Label free	Ratio ^e^ iTRAQ
**gi|15595963**	Serine protease MucD	PA0766	*mucD*	2.86	1.53
**gi|15599785**	Probable outer membrane protein	PA4589	*fadL*	2.51	1.60
**gi|15596964**	Uncharacterized protein	PA1767		2.26	1.77
**gi|15595513**	D-3-phosphoglycerate dehydrogenase	PA0316	*serA*	2.11	1.5
**gi|15598528**	Uncharacterized PhzA/B-like protein	PA3332		2.02	2.23
**gi|15598222**	Uncharacterized protein	PA3026		0.5	0.57

Note:

^a^ Proteins with fold changes of ≥2 or ≤0.5 (label free) and ≥1.5 or ≤0.65 (iTRAQ) are considered significantly more or less abundant in the presence of *n-*octadecane, based on statistic and normalization analysis for four or three biological replicates respectively.

^b^ GI number in accordance with records of *P*. *aeruginosa* PAO1 NCBI database

^c^ Gene products documented in NCBI database and IMG/ER system or annotated on basis of sequence similarities using BLAST tools.

^d^ average ratio from four biological replicates for label free study (C18 /C16)

^e^ average ratio from three biological replicates for iTRAQ study (C18 /C16)

### Functional category of differently expressed proteins

**Fig 2:**
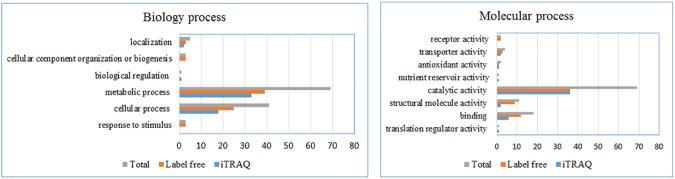
Enrichment analysis results of possible target-related proteins found in label free, iTRAQ, and both two methods.

**Fig 3:**
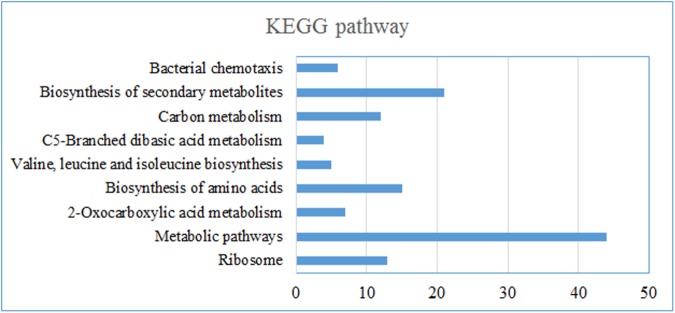
The KEGG pathways in statistically significant differentially expressed proteins of *P*. *aeruginosa* obtained by iTRAQ and label-based methodology.

Those proteins of significant changes by two methods were profiled on basis of GO functional classification and pathway analysis (Fig 2). As shown in Fig 2, for differentially expressed proteins identified by label-free and iTRAQ methods, the most important biology process was “metabolic process”. The proteins belong to metabolic process were analyzed by PANTHER to confirm the pathways being involved. Most of upregulated proteins were involved in serine, threonine, alanine, arginine, valine and isoleucine biosynthesis. However, the downregulated proteins were involved in tryptophan biosynthesis. The second largest section of protein level change was found in “cellular process”. Over half of the proteins have the “catalytic activity” of molecular function (Fig 2). The pathways enrichment was achieved by analyzing the differentially expressed proteins from two methods using string 10.0. As shown in Fig 3, except metabolism and biosynthesis related pathways, “Bacterial chemotaxis” pathway was found to be the most distinct pathway in responds to C18.

### Protein-protein interaction network

We built protein-protein interaction network for those significantly changed proteins from two methods using STRING 10.0 (S1 Fig) [26]. Proteins were grouped into two large units: unit associated with or involved in genetic information processing (left) and bacterial chemotaxis (right). On that condition, we got a clearer insight into the bacterial effect and the hidden relations behind the changes of protein levels in face of C18 environment comparing with C16 by means of a computer-assisted analytical approach.

### Confirmation of the role of FleQ, FliC, NirS, FadL and FadD roles in the n-octadecane acquisition and consumption comparing with n-hexadecane

**Fig 4:**
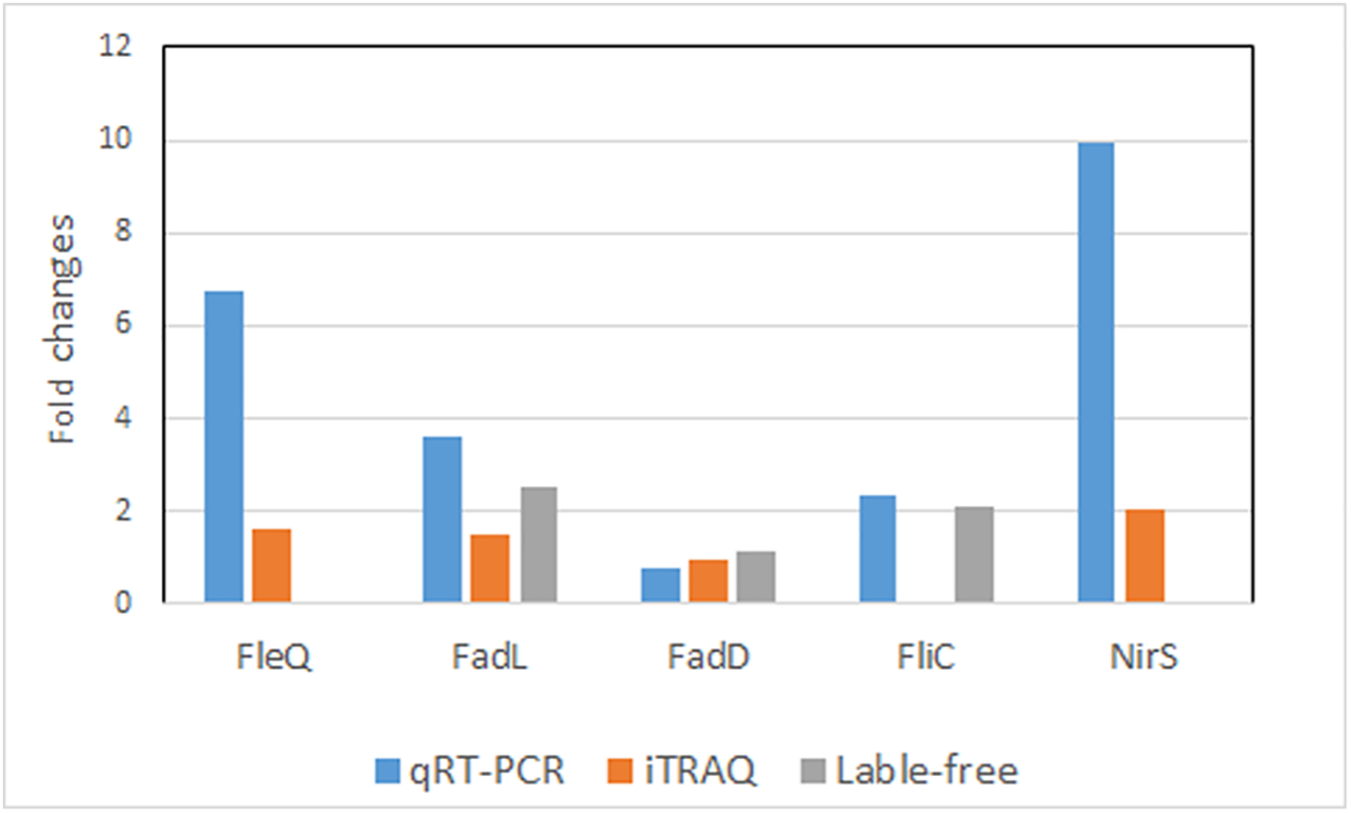
Transcriptional validation of proteomic output using RT-qPCR assays. The value of fold-change in Y-axis indicates the ratio of either protein or mRNA level observed for strain SJTD-1 growing on C18 as opposed to C16 grown cells.

Five genes (FleQ, FliC, NirS, FadL and FadD) from *P*. *aeruginosa* SJTD-1 were selected for qRT-PCR analysis to quantify their transcriptional levels. As shown in Fig 4, transcripts of FleQ, FliC, NirS and FadL genes were significantly different comparing C18-grown cells with C16-grown cells. Despite the different fold changes obtained from qRT-PCR and proteomics, the overall trends of gene expression are consistent. FadD has no change in transcriptional levels, which is the same with the results of comparative proteomics analysis (S2 and S4 Tables). In all, results of qRT-PCR means the agreement with those of proteomics.

## Discussion

### iTRAQ and label-free quantitative proteomic analysis

The quantification proteomics methods of label-free and iTRAQ have been published in several studies [28–31]. It has been indicated that each method had its advantages and disadvantages by comparison of these two methods in previous reports. In order to study the different expressed proteins depending on n-octadecane comparing with n-hexadecane, iTRAQ and label-free quantitative proteomic analysis were both conducted to identify the proteins in *P*. *aeruginosa* SJTD-1. In our study, the number of proteins identified in label free analysis (1249 proteins) was slightly higher than that of iTRAQ based analysis (1102 proteins), while the fact that there were 907 common proteins identified in both methods suggested the consistence of both methods.

### Enrichment and protein-protein interaction network analysis

Bioinformatics analysis was conducted to analyze the differential expressed proteins found in quantification proteomic analysis. Enrichment analysis results showed that “metabolic process” and “cellular process” were the most important biology process. Interestingly, despite of the difference in number of proteins, enrichment analysis results based on differential expressed proteins found in label-free and iTRAQ-based method were consistent with each other. Interestingly, most of upregulated proteins were involved in serine, alanine, arginine or other amino acid biosynthesis except tryptophan biosynthesis in which the downregulated proteins were involved. Phosphorylated enolpyruvate, offered by gluconeogenesis pathway, was demanded in the synthesis of tryptophan. Yet, the synthesis of serine, alanine, arginine or other amino acid was related with fatty acid metabolism, glycolysis and tricarboxylic acid cycle. It would be speculated that the C16-cultivatd stains has more usage efficiency of n-hexadecane than C18-cultivated stains with n-octadecane by the inhibited gluconeogenesis pathway and increased serine, alanine, arginine or other amino acid biosynthesis. “Bacterial chemotaxis” was found to be the important pathway, of which the proteins included showed the strong interaction among them by protein-protein interaction network analysis. Chemotaxis pathways have been found to mediate flagellum- and type IV-pili mediated motility, playing important role in access compounds that serve as carbon/nitrogen sources or electron acceptors [22,32]. In sum, the results of comparative proteomics suggested chemotaxis still play an important role in responds to n-octadecane comparing with n-hexadecane in order to acquire alkanes of longer carbon chain.

### Swimming performance of *P*.*aeruginosa* SJTD-1

The study before has shown that n-hexadecane is the greatest substrate of *P*.*aeruginosa* SJTD-1, followed by n-octadecane [7]. It has been proposed that the rapid depletion of *n-*alkanes by microorganisms may be driven by two factors: the activity of monooxygenases for oxidation of n-alkanes and the transport of alkanes into the cells [33]. Our previous results have proved that the increased expression ratio of monooxygenases for oxidation of *n-*alkanes (C12-C24) was largely different for *P*. *aeruginosa* SJTD-1 to maintain the greatest depletion rate [19]. Yet the degradation rates of alkanes by monooxygenases exceed the rates of diffusive flux into microbial cells and dissolution into the aqueous phase [34–36]. Thus, the uptake rates of alkane may play the key role in determining the rates of alkanes assumption.

n-Octadecane have the much higher hydrophobicity than n-hexadecane. It has been reported that the pseudo solubilized rates of n-octadecane is lower (1.91±0.17mg) than n-hexadecane (2.63±0.21 mg) at 120 h [37]. In order to improve the capture of n-octadecane, some strategies have been used to increase the swimming performance to overcome the low solubility of n-octadecane and enhance transport of bacteria. In our study, FleQ, FliC and NirS proteins have been demonstrated to be upregulated in responds to n-octadecane comparing with control (C16-grwon cells) (S3 and S5 Tables, Fig 4). FleQ is NtrC/NifA-type transcriptional activator, which is identified to be involved in flagellar gene expression [38]. FliC and NirS have been found play an important role in flagellum formation and motility [39]. Thus, the flagellar genes would be over-expressed by the upregulated FleQ, as well as NirS and FliC proteins, which would lead to the strengthened swimming performance of *P*. *aeruginosa* SJTD-1 being benefit to capture n-octadecane with higher hydrophobicity than n-hexadecane.

### n-Octadecane transport

Recently, FadL has been characterized in *E*. *coli* to specifically recognize and transport not only the long-chain fatty acids, but also the medium-chain alkane [19,21]. In our previous research, we also proposed that the FadL and FadD would play an important role in uptake alkanes in *P*. *aeruginosa* [22]. In this comparative proteomics analysis, FadL has an abundance expression by both label free and iTRAQ-based methods comparing cells cultivated on C18 with C16, and this result has been further confirmed by qRT-PCR. Thus, our results not only further demonstrated that FadL was very important in uptake alkane into cells in *P*. *aeruginosa*, but also proposed that the longer the chain alkane, the more requirement the FadL transport protein. In our study, FadD showed no increment during C18 growth by two methods, suggesting that although the expression of FadD is related with alkane growth [22,40], the length of carbon chain may have no influence on its expression.

## Concluding remarks

In conclusion, it is benefit to identify uptake proteins of differential expression comparing the C18-cultivated stains with C16-cultivated stains in *P*. *aeruginosa* SJTD-1 by iTRAQ and label-free comparative proteomics analysis. 91 and 99 proteins showing different abundance comparing SJTD-1 cultivated on C18 with C16 were identified by label-free and iTRAQ comparative proteomics analysis respectively. The number of common differentially expressed proteins of both methods was 6 proteins. Specifically, our analysis evidenced that bacterial chemotaxis was the important pathway in responds to n-octadecane. Moreover, five genes (FleQ, FliC, NirS, FadL and FadD) from *P*. *aeruginosa* SJTD-1 were confirmed to be abundance on C18. Further studies are needed to determine the significance of these proteins, but overall these data provide a solid foundation for alkane consumption mechanism for future.

## Supporting information

S1 Fig Possible signal network and distinct pathway related to the effects of C18. Network established using STRING 10.0 based on the significantly changed proteins found in both label-free and iTRAQ-based methods. Lines indicate known or predicted protein-protein interactions, with purple lines indicating the interaction are experimentally determined.
(PNG)

S1 Table List of primers used in this work. All primers are listed from 5’ to 3’. The F and R primers represent forward and reverse primers, respectively.
(DOCX)

S2 Table Proteins report for *P*. *aeruginosa* SJTD-1 by iTRAQ-based method. (XLSX)

S3 Table All significant different expression proteins for *P*. *aeruginosa* SJTD-1 by iTRAQ based method. Proteins with fold changes of ≥ 1.5 or ≤ 0.65 are considered significantly more or less abundant in the presence of n-octadecane, respectively, based on statistic and normalization analysis for three biological replicates. Accession number in accordance with records of P. aeruginosa PAO1 NCBI database. Fold: average ratio from three biological replicates for iTRAQ study (C18 / C16).
(XLSX)

S4 Table Proteins report for *P*. *aeruginosa* SJTD-1 by label free-based method. (XLSX)

S5 Table All significant different expression proteins for *P*. *aeruginosa* SJTD-1 by label free- based method. Proteins with fold changes of ≥2 or ≤ 0.5 are considered significantly more or less abundant in the presence of n-octadecane, respectively, based on statistic and normalization analysis for four biological replicates. Accession number in accordance with records of *P*. *aeruginosa* PAO1 NCBI database. Fold: average ratio from four biological replicates for label free study (C18 / C16).
(XLSX)

## Abbreviations

C18: N-octadecane
C16: N-hexadecane
qRT-PCR: Quantitative reverse transcriptase PCR
