# Supplementary figures and images for: Quantitative proteomics analysis of proteins involved in alkane uptake comparing the profiling of Pseudomonas aeruginosa SJTD-1 in response to n-octadecane and n-hexadecane
Source: PLoS One. 2017 Jun 29;12(6):e0179842. doi: 10.1371/journal.pone.0179842 (PMC5491041; doi:10.1371/journal.pone.0179842)

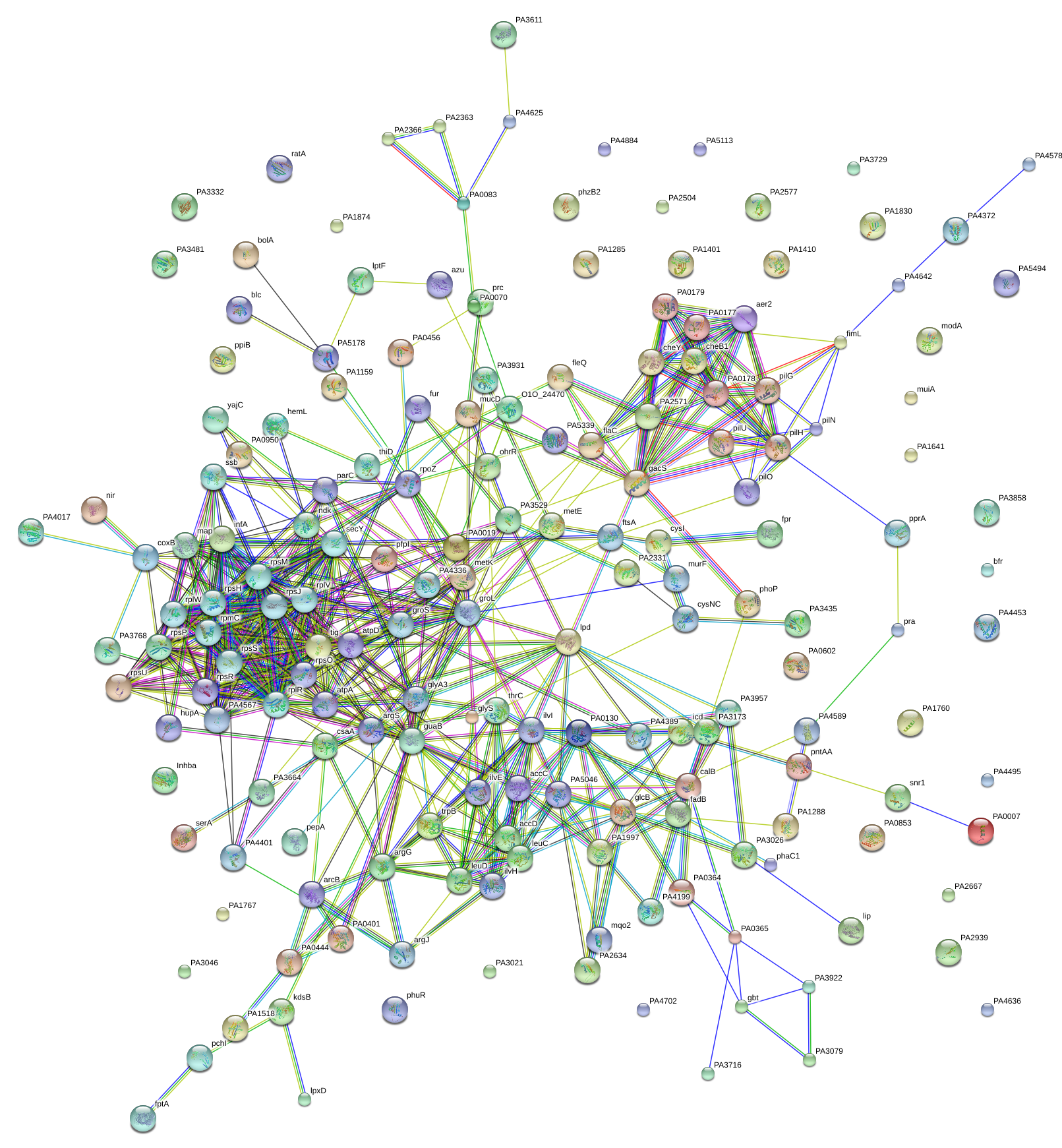

Supplement: S1 Fig — Network established using STRING 10.0 based on the significantly changed proteins found in both label-free and iTRAQ-based methods. Lines indicate known or predicted protein-protein interactions, with purple lines indicating the interaction are experimentally determined. (PNG) [file pone.0179842.s001.png]
