# Supplementary material for: Quantitative proteomics analysis of proteins involved in alkane uptake comparing the profiling of Pseudomonas aeruginosa SJTD-1 in response to n-octadecane and n-hexadecane
Source: PLoS One. 2017 Jun 29;12(6):e0179842. doi: 10.1371/journal.pone.0179842 (PMC5491041; doi:10.1371/journal.pone.0179842)
Supplement: S1 Table — All primers are listed from 5’ to 3’. The F and R primers represent forward and reverse primers, respectively. (DOCX) [file pone.0179842.s002.docx]

**Table S1 List of primers used in this work**

| Name | Sequence^*^ |
| --- | --- |
| 16S-F | AACTGAAGAGTTTGATCATGGCTCAG |
| 16S-R | AGATTCCTAGGCATTACTCACCC |
| FleQ-F | aggattggcgagaggatgcgc |
| FleQ-R | tcagcaggatcggcaggtactc |
| Flic-F | GGTCAGGTCGCAGTGAAGGTCC |
| Flic-R | GAGTTCAGTTGCACGTAGCCGG |
| NirS-F | AAGGACGACATGAAAGCCG |
| NirS-R | TTGGAAGTAGATCTGCTTGGC |
| FadL-F | GGCTCGCTGTTTCCCAAC |
| FadL-R | AGGCATCGCCGTTGTACT |
| FadD-L | ATGCAACCTGAATTCTGGAACG |
| FadD-R | AATTTCTTGCAGGAGCGCT |

^*^ All primers are listed from 5’ to 3’. The F and R primers represent forward and reverse primers, respectively.
